# In Vitro and *In Silico* Evaluation of Isatin‐Derived Spirooxindoles as Antituberculosis Drug Candidates

**DOI:** 10.1111/cbdd.70152

**Published:** 2025-07-01

**Authors:** Fernanda Rodrigues de Lima, Jéssika de Oliveira Viana, Aleff Cruz de Castro, Rodrigo Cristiano, Marcia Alberton Perelló, Alexia de Matos Czeczot, Cristiano Valim Bizarro, Pablo Machado, Luiz Augusto Basso, Claudio Gabriel Lima‐Junior, Valnês da Silva Rodrigues‐Junior, Karen Cacilda Weber

**Affiliations:** ^1^ Programa de Pós‐Graduação em Química Universidade Federal da Paraíba (UFPB) João Pessoa Brazil; ^2^ National Institute of Science and Technology on Molecular Sciences‐INCT‐CiMol João Pessoa Brazil; ^3^ Centro de Pesquisas em Biologia Molecular e Funcional (CPBMF), Instituto Nacional de Ciência e Tecnologia em Tuberculose (INCT‐TB) Pontifícia Universidade Católica do Rio Grande do Sul (PUCRS) Porto Alegre Brazil; ^4^ Programa de Pós‐Graduação em Biologia Celular e Molecular, Escola de Ciências da Saúde e da Vida, PUCRS Porto Alegre Brazil; ^5^ Programa de Pós‐Graduação em Medicina e Ciências da Saúde, PUCRS Porto Alegre Brazil; ^6^ Programa de Pós‐Graduação em Produtos Naturais e Sintéticos Bioativos Universidade Federal da Paraíba (UFPB) João Pessoa Brazil

**Keywords:** antituberculosis agents, inverse docking, isatin‐derivatives, multidrug‐resistant *Mycobacterium tuberculosis*, spirooxindoles

## Abstract

Tuberculosis (TB) remains a major global health threat, exacerbated by multidrug‐resistant *Mycobacterium tuberculosis* (MTB) strains. The development of new anti‐TB agents is crucial. In this study, 17 isatin derivatives synthesized by our research group were evaluated for their in vitro activity against MTB strains and the two most potent compounds were assessed for cytotoxicity. Additionally, molecular docking was performed against 22 MTB protein targets to explore possible mechanisms of action, and ADMET predictions were used to determine pharmacokinetic and pharmacodynamic suitability. Also, we investigated the activity of A15, A16, and A17 against two genetically characterized multidrug‐resistant clinical isolates (PT‐12 and PT‐20). As a result, the compounds A16 and A17 exhibited the highest anti‐TB activity (MIC = 10 μM for both). Inverse molecular docking indicated the enzyme enoyl‐[acyl‐carrier‐protein] reductase as a potential biological target. Cytotoxicity assays confirmed that A16 and A17 were non‐toxic, and ADMET predictions indicated suitable drug‐like properties for anti‐TB therapy. Notably, A16 and A17 showed inhibitory effects against drug‐resistant MTB isolates, with minimum inhibitory concentrations (MICs) ranging from 10 to 20 μM, suggesting their potential to overcome resistance mechanisms linked to mutations in katG and rpoB. These findings highlight A16 and A17 as promising candidates for anti‐TB agents, particularly against multidrug‐resistant strains.

## Graphical abstract

Two compounds, A16 and A17, were active against multidrug‐resistant *Mycobacterium tuberculosis* isolates (PT‐12 and PT‐20), overcoming key resistance mutations in katG and rpoB, while cytotoxicity assays confirmed that they are non‐toxic. A17 exhibited the highest antituberculosis activity, with molecular docking suggesting enoyl‐[acyl‐carrier‐protein] reductase as a potential biological target.

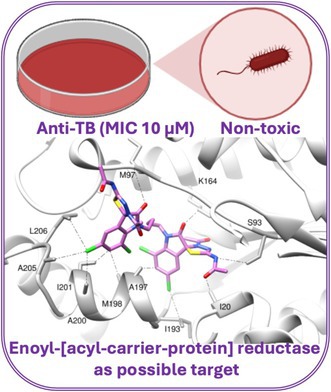

## 1 Introduction

Tuberculosis (TB) remains a major global health challenge, particularly in low‐income populations. The disease is caused by *Mycobacterium tuberculosis* (MTB), a highly adaptable pathogen with a robust cell wall and the ability to persist in the host for prolonged periods (Singh et al. 2017). Despite extensive control efforts, TB incidence continues to grow considerably today. According to the latest Global Tuberculosis Report from the World Health Organization, an estimated 10.8 million people fell ill with tuberculosis (TB) in 2023, resulting in approximately 1.25 million deaths. This makes TB the leading cause of death from infectious diseases in 2023, surpassing COVID‐19 (WHO 2024). The emergence of drug‐resistant TB strains further underscores the urgent need for novel therapeutic agents.

TB treatment may vary depending on the body's resistance to the drugs used. Typically, treatment with these medications lasts 6 months and is applied in a basic scheme consisting of Rifampicin, Isoniazid, Pyrazinamide and Ethambutol (RHZE) for 2 months and (Rifampicin+Isoniazid) for 4 months (WHO 2025). However, the prolonged duration and complexity of using multiple medication regimens lead to unsatisfactory treatment adherence, which can contribute to the development of drug‐resistant TB strains (Dheda et al. 2024). Rifampicin and Isoniazid have different inhibition mechanisms, but the development of resistant strains is already observed in both cases. For Rifampicin, the development of resistance occurs with mutations in the central region of the gene that encodes the β subunit of RNA polymerase, while with Isoniazid, mutations occur in the regulatory regions of the InhA gene (The CRyPTIC Consortium 2022).

Therefore, it is crucial to discover new classes of drugs with anti‐TB activity. Among the most promising compounds studied recently, we can highlight the derivatives of isatin, an organic molecule that has a nucleus with an extensive range of substitution possibilities, giving rise to several derivatives with interesting chemical and physical properties (Nath et al. 2020; Sridhar et al. 2024; Nalini et al. 2023; Santos et al. 2023). Pre‐clinical and clinical reported data are available demonstrating that isatin derivatives present diverse biological activities such as anticancer, anti‐TB, antifungal, antimicrobial, anticonvulsant, and anti‐HIV (Cheke et al. 2022). Among the FDA‐approved drugs containing an isatin scaffold, sunitinib stands out as a multi‐targeted tyrosine kinase inhibitor used in the treatment of renal cell carcinoma, gastrointestinal stromal tumors, and pancreatic neuroendocrine tumors (Blumenthal et al. 2012). Another example is nintedanib, which has been approved for idiopathic pulmonary fibrosis (Richeldi et al. 2014) and non‐small‐cell lung cancer (Popat et al. 2015). In the veterinary field, toceranib is approved for the treatment of canine mastocytoma (London et al. 2009).

An example of promising isatin derivatives are the spirocyclic compounds. The term “spirocycle” or “spirocompound” is used to describe a family of compounds that have an sp^3^‐hybridized carbon atom linked to two cycles simultaneously. Therefore, these compounds are of great interest to researchers in both synthetic organic chemistry and medicinal chemistry, as this characteristic is common in many natural products that have an indole nucleus and are used to construct pharmacologically active compounds (da Costa et al. 2021). Spirocyclic compounds containing the indole nucleus showed antimalarial, antimicrobial, and anticancer activity (Singh and Desta 2012; da Costa et al. 2021; Castro et al. 2023). Therefore, such molecules can be used as promising nuclei for drug candidates, as their derivatives participate in biochemical processes in mammals, with desirable pharmacokinetic profiles and simple, cheap, and sustainable production.

Despite some in vitro studies involving isatin derivatives that have already shown potential for antituberculosis activity (Jiang et al. 2018), the investigation of the mechanisms of action of these drug candidates in the TB pathology is still necessary. For this, it is crucial to identify possible biological receptors for these molecules. However, the experimental determination of biological targets involves expensive and complex techniques, which require, for example, the isolation, expression, and purification of proteins, as well as mass spectroscopy analyses, among others. A promising alternative to overcome these limitations is the identification of targets using computational methods (Galati et al. 2021). In the target identification problem, there are two categories of computational strategies that can be used: those based on the structure of ligands, which use similarity search and machine learning techniques, and those based on the structure of the receptor, which essentially employ molecular docking or pharmacophoric search techniques (Ziegler et al. 2010). In general, with advances in computational technologies, computer‐aided drug design (CADD) techniques constitute important tools that have emerged in order to optimize the process of discovering new drugs (Yu and Mackerell 2017).

In this work, aiming to identify promising candidates for further development as anti‐TB agents, a series of 17 spirooxindoles previously synthesized by our research group (da Costa et al. 2021; Castro et al. 2023) have been tested in vitro against MTB strains and multi‐drug resistant clinical isolates of *M. tuberculosis* to evaluate their anti‐TB activity. In vitro cytotoxicity of the two most potent compounds was also investigated. Additionally, in order to identify possible biological targets related to the antituberculosis activity experimentally observed, we applied an inverse docking target fishing approach, by performing molecular docking of the three most potent compounds on 22 MTB protein targets with structures available in the RCSB Protein Data Bank (PDB). Through this strategy, it was possible to suggest a putative inhibition mechanism for the ligands under study. Furthermore, a computational evaluation of the ADMET properties of these ligands was carried out in order to complement the analyses. Our findings suggest a good pharmacodynamic and pharmacokinetic profile for the compounds under investigation.

## 2 Methods

### 2.1 Chemical Structures

**FIGURE 1:**
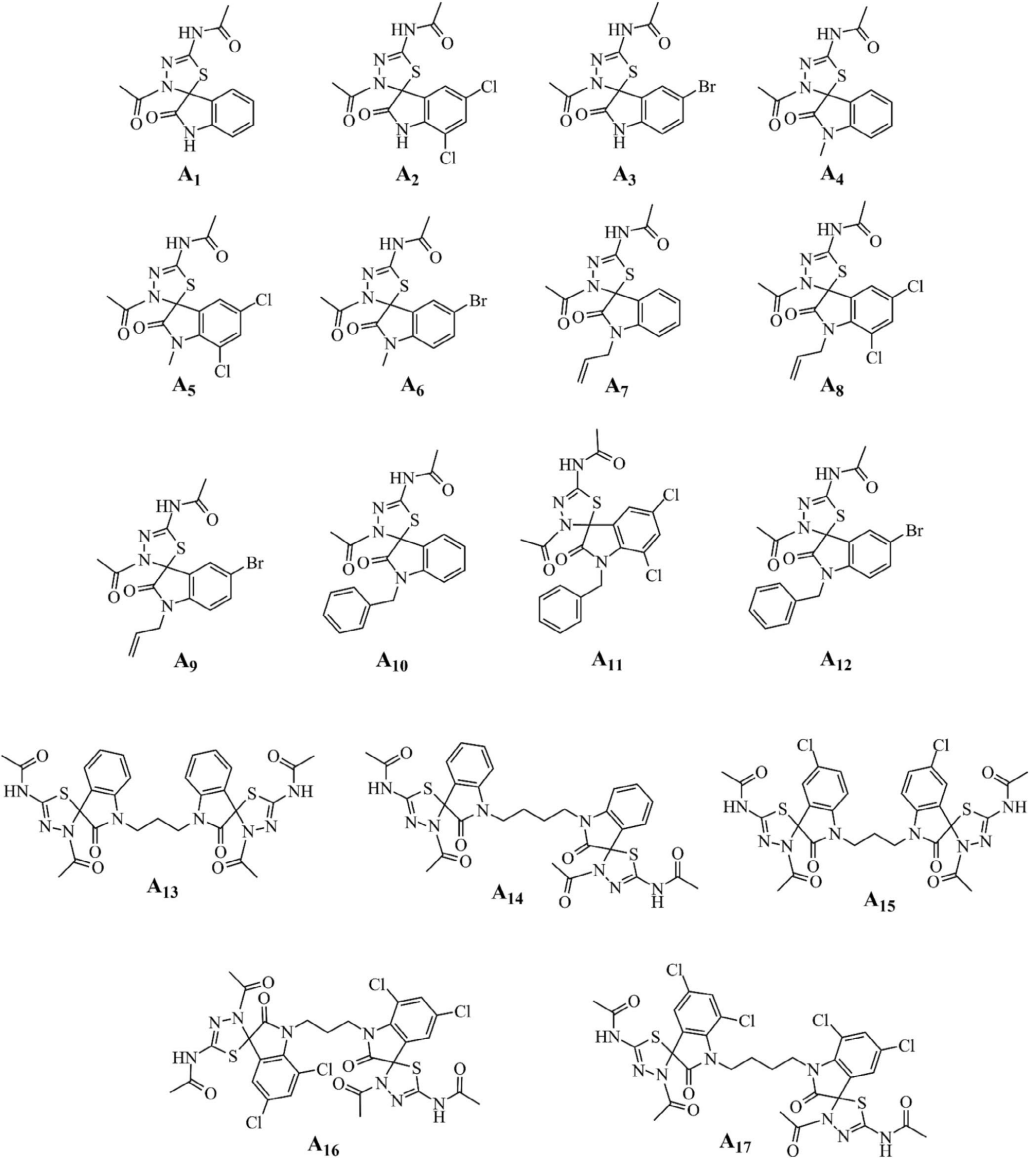
Chemical structures of the compounds under investigation.

Rifampicin, isoniazid, and moxifloxacin were purchased from Sigma‐Aldrich. Isatin‐derived spirooxindoles were synthesized as previously described (da Costa et al. 2021; Castro et al. 2023). The chemical structures of 17 compounds of the series under study are shown in Figure 1. While the sp^3^ spiro‐C structure introduces a chiral center to the proposed spirooxindoles, the synthesis was performed without controlling the stereochemistry (da Costa et al. 2021). As a result, the dimeric compounds consist of a stereoisomeric mixture of two enantiomers (1S, 2S; 1R, 2R) and the meso compound (Castro et al. 2023). Screening for anti‐mycobacterial potential, determination of MIC against virulent and resistant *M. tuberculosis* and cytotoxicity investigation procedures are detailed in Supporting Information S1.

### 2.2 Molecular Docking Procedure

#### 2.2.1 Ligand Dataset Preparation

**FIGURE 2:**
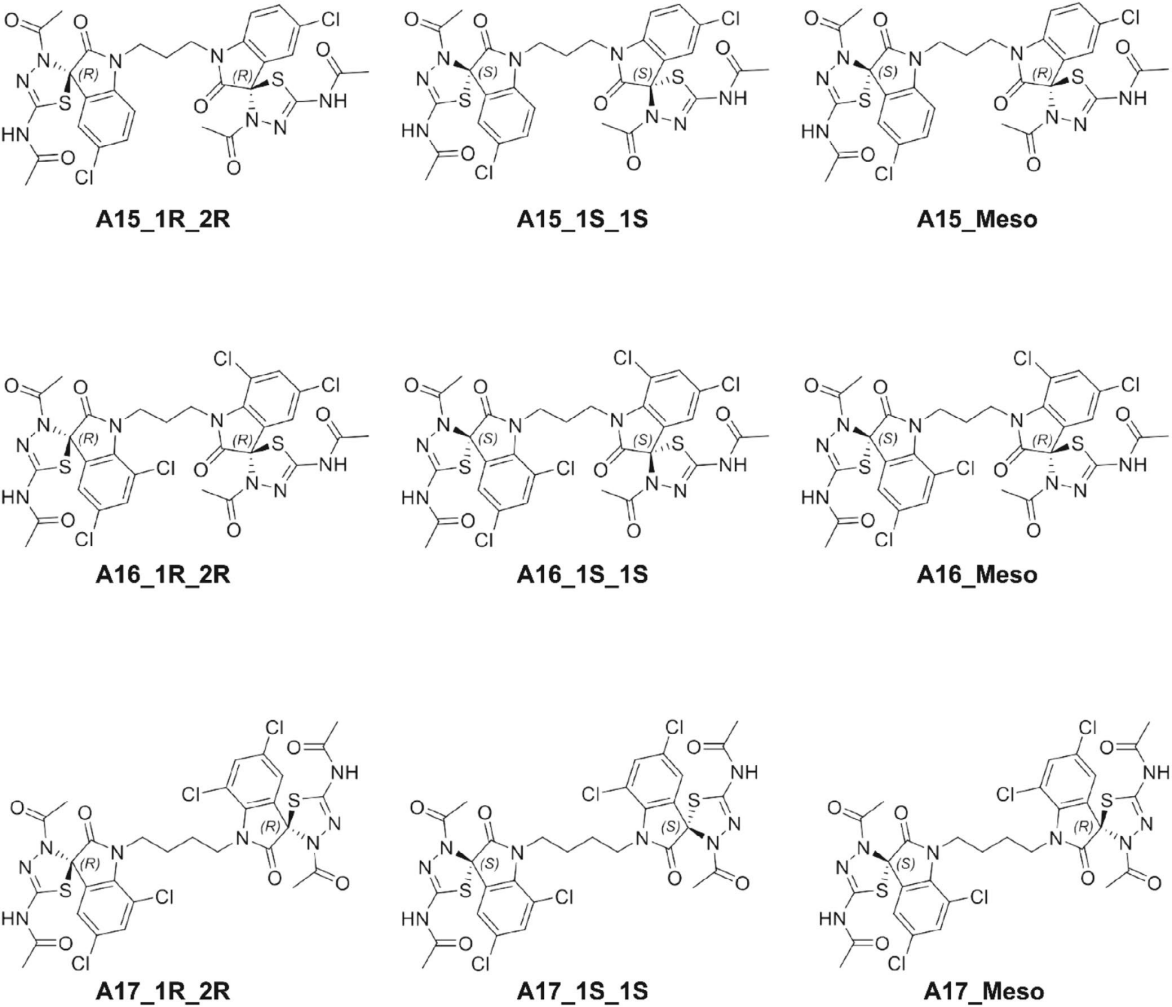
Structural representations of the 1R_2R and 1S_2S enantiomers and the 1S_2R meso compounds of A15, A16, and A17.

The three most potent against MTB isatin‐derived spirooxindoles, synthesized by (Castro et al. 2023), A15, A16, and A17, were modeled in Marvin Sketch 23.10 (ChemAxon 2023). Each of the structures has two asymmetric carbons, so a pair of enantiomers and a meso compound were identified, as shown in Figure 2, resulting in nine structures for the *in silico* analyses. In order to obtain optimized conformations for the molecules, energy minimization was carried out by using Openbabel (O'Boyle et al. 2011), consisting of 1500 steps with the steepest descent algorithm.

#### 2.2.2 Protein Preparation

From a literature search, 22 MTB proteins were selected to perform docking tests to estimate the best match between the nine possible stereoisomers of each ligand with all protein targets in the dataset. Some of the MTB target structures in the PDB database (Berman et al. 2000) are complexed with drugs already available for commercial use and others in advanced stages of clinical trials. Table 1 presents the PDB codes and the protein function reported in the literature.

**TABLE 1:** PDB codes and functions of the MTB targets selected to compose the data set.

PDB code	Protein name	Metabolic pathway
1ENY	Enoyl‐[acyl‐carrier‐protein] reductase	Mycolic acid synthesis
2H9I	Enoyl‐[acyl‐carrier‐protein] reductase	Mycolic acid synthesis
2 IE0	Enoyl‐[acyl‐carrier‐protein] reductase	Mycolic acid synthesis
4BGE	Enoyl‐[acyl‐carrier‐protein] reductase	Mycolic acid synthesis
4BGI	Enoyl‐[acyl‐carrier‐protein] reductase	Mycolic acid synthesis
4BII	Enoyl‐[acyl‐carrier‐protein] reductase	Mycolic acid synthesis
4OHU	Enoyl‐[acyl‐carrier‐protein] reductase	Mycolic acid synthesis
3Q0V	HTH‐type transcriptional regulator EthR	Mycolic acid synthesis
5MXV	HTH‐type transcriptional regulator EthR	Mycolic acid synthesis
5MYN	HTH‐type transcriptional regulator EthR	Mycolic acid synthesis
5MYR	HTH‐type transcriptional regulator EthR	Mycolic acid synthesis
5MYS	HTH‐type transcriptional regulator EthR	Mycolic acid synthesis
5MYT	HTH‐type transcriptional regulator EthR	Mycolic acid synthesis
5MYW	HTH‐type transcriptional regulator EthR	Mycolic acid synthesis
3R5L	Deazaflavin‐dependent nitroreductase	Mycolic acid synthesis
3IFZ	DNA gyrase subunit A	DNA supercoiling
3ZKD	DNA gyrase subunit B	DNA supercoiling
1EYE	Dihydropteroate synthase I	Folate biosynthesis
1XFC	Alanine racemase	Peptidoglycan synthesis
3PTY	Arabinosyltransferase C	Arabinogalactan biosynthesis
4V1F	F0F1 ATP synthase subunit C	ATP biosynthesis
5UHG	DNA‐directed RNA polymerase subunit alpha	Transcription initiation

Protein structures preparation was carried out in the DockPrep module of the UCSF Chimera 1.17.3 program (Pettersen et al. 2004). Bound ligands and water molecules were removed, hydrogen atoms were added to the structure and the protonation states were assigned with the H++ server (Dhanoa et al. 2011) at a physiological pH of 7.4.

In the case of more than one equal protein subunit in a given PDB entry, the preference was to use only the A subunit. The enzymes with PDB codes 1EYE, 1XFC, 3IFZ, 3PTY, 3ZKD, and 4BII have missing amino acid residues, which were completed by comparative modelling using the Swiss‐Model server (Waterhouse et al. 2018). The best three‐dimensional models obtained were analyzed and their reliability was evaluated using Molprobity parameters and the Ramachandran plots (Chen et al. 2010).

#### 2.2.3 Molecular Docking of Compounds in MTB Targets

Molecular docking calculations of the nine stereoisomers of compounds A15, A16, and A17 on the 22 selected enzymes were performed in the GOLD 2022.3.0 program (Jones et al. 1997) using a radius of 25 Å and the four scoring functions, ASP, ChemPLP, ChemScore, and GoldScore, to explore different aspects of protein‐ligand interactions and improve the overall accuracy of docking predictions. A consensus docking analysis was carried out using the rank‐by‐number consensus strategy (Wang and Wang 2001), consisting of normalizing the docking scores by dividing each ligand‐receptor score by the highest score amongst them and then calculating the average normalized score over all scoring functions. The highest value was considered as indicating the best match between ligand stereoisomers and MTB targets.

#### 2.2.4 Redocking Protocol

After visual inspection and molecular docking of the structures of MTB molecular targets, the target with the best interactions with the compounds was selected to perform redocking tests. In these tests, the crystallized ligand was removed from the binding site and then docked in GOLD, to compare its results with the crystallographic pose. The molecular docking follows the best protocol established in the previous docking step. The analyses of the redocking results were carried out in Pymol to calculate the Root Mean Square Deviation (RMSD), the parameter used to compare the pose of the docked molecule with the co‐crystallized one.

#### 2.2.5 ROC Curve Calculation

The molecular target with PDB code 4OHU, with 1.60 Å resolution, was selected for calculating the ROC curve because it is the only one of Enoyl‐[acyl‐carrier‐protein] reductase enzymes, in our dataset, that has experimental data of inhibition constant (*K*_i_) available in the ChEMBL database (Gaulton et al. 2012). In this, 60 ligands that had reported *K*_i_ values showing inhibition potential for this enzyme were selected. The lower the value obtained, the better the result of the biological test. The results for this search were exported to an Excel spreadsheet. Compound screening involved removing duplicates and adjusting for ionic regions in the SMILES code. In total, 45 compounds categorized as active (*K*_i_ ranging from 129 to 0.2 nM) and 15 as inactive (*K*_i_ ranging from 71,000 to 130 nM) were identified. The SMILES codes of the active compounds were employed to produce the decoys using the DUD‐E platform (Mysinger and Shoichet 2010). For each active compound in the series, a set of 51 decoys was generated. Subsequently, the three‐dimensional structures of the active and inactive compounds, along with the decoys, were constructed using OpenBabel. This process involved setting a pH of 7.4, generating the 3D structure and employing hydrogen bonds as standardization criteria. The resulting compounds were saved in mol2 format for use in the GOLD program.

With the compound structures in hand, molecular docking was performed for the active, inactive, and decoy compounds using grid box coordinates and the protein structure with the same parameters used in redocking. The score values were calculated and a spreadsheet was generated containing the names of the compounds, their corresponding scores, and a third column assigning the value 1 for active compounds and 0 for inactive compounds and decoys. The ROC curve was calculated using the server available at https://stats.drugdesign.fr/ (Kuhn and Sander 2013) where values of AUC (area under curve) close to 1 indicate a more reliable model prediction.

### 2.3 ADMET Properties Calculation

In order to investigate the Absorption, Distribution, Metabolism, Excretion, and Toxicity (ADMET) properties of the compounds, the following pharmacokinetic parameters were calculated: water solubility, octanol–water partition coefficient (logP), molecular weight, number of hydrogen acceptors, number of hydrogen donors and Blood Brain Barrier (BBB) permeability in SwissADME (Daina et al. 2017), and also AMES toxicity, gastrointestinal (GI) absorption, protein inhibition and P‐glycoprotein (P‐gp) interaction, in the pkCSM server (Pires et al. 2015).

## 3 Results and Discussion

### 3.1 Isatin‐Derived Spirooxindoles Are Active Against *M. tuberculosis* Laboratory Strains

We first determined the antimycobacterial potential for A1, A2, A3, A4, A5, A6, A7, A8, A9, A10, A11, A12, A13, A14, A15, A16, and A17 against *Mycobacterium tuberculosis* H37Ra. The MIC for A5, A8, and A9 was found to be 200 μM, and for A11, 100 μM. All other evaluated molecules A1, A2, A3, A4, A6, A7, A10, A12, A13, A14 did not inhibit *Mycobacterium tuberculosis* H37Ra growth at the maximum concentration tested, 200 μM, so their MICs were estimated to be higher than 200 μM. As presented in Table 2, A15, A16, and A17 suppressed the growth of *M. tuberculosis* H37Ra with MIC values lower than 40 μM. The MICs found for the standard therapeutic agents utilized in these assays, rifampicin and moxifloxacin, were 0.03 and 0.2 μM, respectively.

**TABLE 2:** Activity of isatin‐derived spirooxindoles drug candidates against *M. tuberculosis* .

Compounds	MIC (μM)^a^			
	*M. tuberculosis* H37Ra	*M. tuberculosis* H37Rv	PT‐12^b^	PT‐20^b^
A15	40	80	40	40
A16	10	40	20	20
A17	10	20	20	10

^a^ MIC values reported here were observed in three or four independent experiments.

^b^ Multi‐drug resistant PT‐12 and PT‐20 clinical isolates hold mutations in katG (S315T) and rpoB (S531L) genes.

A15, A16, and A17 were consequently chosen for additional evaluation using the virulent laboratory H37Rv strain of *M. tuberculosis* . A17 was the more potent molecule, with MICs of 20 μM (Table 2). The values reported here were observed in three independent experiments or were the highest found among three independent tests.

### 3.2 Isatin‐Derived Spirooxindoles Are Active Against Multi‐Drug Resistant Clinical Isolates of *M. tuberculosis*

In parallel, we analyzed the impacts of A15, A16, and A17 against drug‐resistant clinical isolates of *M. tuberculosis* . Two multi‐drug resistant clinical isolates (PT‐12 and PT‐20), genetically defined earlier (Perdigão et al. 2014), were evaluated. Multi‐drug resistant PT‐12 and PT‐20 carry the mutation (S531L) in the *rpo*B gene, responsible for causing resistance to rifampicin, and also carry the mutation (S315T) in the *kat*G (Rv1908c) gene, which is the most frequent mutation found in isoniazid‐resistant strains. Of note, A16 and A17 affected the viability of drug‐resistant isolates of *M. tuberculosis* with MIC values ranging from 10 to 20 μM (Table 2). The MIC values found for isoniazid were 146 μM for PT‐12 and 292 μM for PT‐20. For rifampicin, the MIC values were higher than 80 μM for both clinical isolates, PT‐12 and PT‐20. These results indicate that A16 and A17 could be able to overcome the main resistance mechanisms found in resistant clinical isolates of *M. tuberculosis* , such as genetic modifications in *kat*G or *rpo*B genes.

### 3.3 Cytotoxicity Evaluation of A16 and A17 on Vero E6 Cell Viability

**FIGURE 3:**
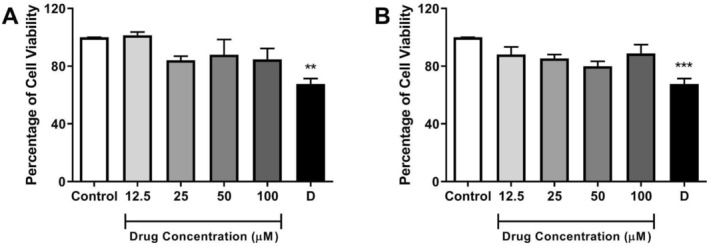
Data of cytotoxic effects of test compound A16 (A) and A17 (B) on Vero E6 cells. Control: 1% DMSO‐treated wells, considered as 100% cell viability; D: 10% DMSO. Data were expressed as mean of cell viability ± standard error of mean of three to four independent experiments performed in triplicate. ***p < 0.001, **p < 0.01, compared to the corresponding control group.

We have investigated the possible in vitro cytotoxic effects of compounds A16 and A17 using the MTT assay. African green monkey kidney (Vero) cells were used in these experiments. Cellular viability was evaluated after exposition of the cell lineage with the compounds (A16 and A17) for 72 h. The results were expressed as a percentage of cell viability, considering the 1% DMSO‐treated control wells as 100% of cell viability. The in vitro incubation of the compounds A16 and A17, at concentrations ranging from 12.5 to 100 μM, did not significantly affect the cell viability of this eukaryotic cell line (Figure 3), indicating a favorable cytotoxicity profile within this concentration range.

### 3.4 Selection of Best Ligand‐Receptor Complexes by Consensus Docking

Different scoring functions employ distinct mathematical models and assumptions, leading to variability in their predictions. Some scoring functions prioritize enthalpic contributions, such as hydrogen bonding and electrostatic interactions, while others emphasize hydrophobic effects or empirical parameters derived from experimental binding data. Given these methodological differences, relying on a single scoring function may introduce bias or fail to capture the full complexity of ligand‐receptor interactions. To improve the reliability of target selection, we employed multiple scoring functions and applied the rank‐by‐number method, which ranks targets based on the average predicted values given by all the scoring functions. This consensus‐based strategy reduces the influence of outlier results from individual scoring functions and provides a more robust assessment of which TB protein targets exhibit the strongest and most consistent ligand binding, ultimately improving the confidence in target prediction.

**FIGURE 4:**
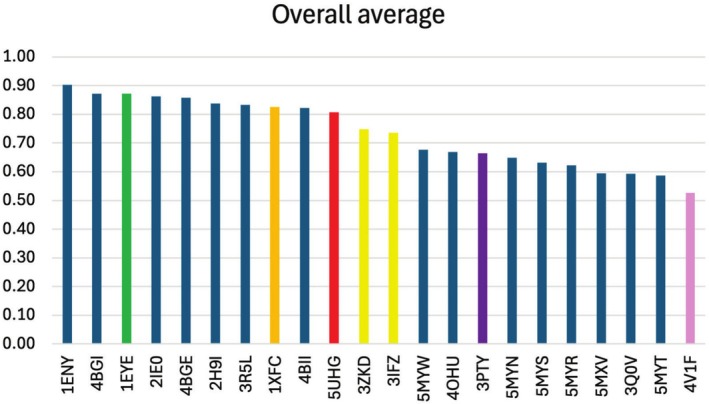
Overall average of docking scores in the four scoring functions for the TB targets analyzed. Different colors represent different metabolic pathways (blue for mycolic acid synthesis, green for folate biosynthesis, orange for peptidoglycan synthesis, red for transcription initiation, yellow for DNA supercoiling, purple for arabinogalactan biosynthesis, and light pink for ATP biosynthesis).

Docking scores for all scoring functions are presented in Supporting Information S2. The rank‐by‐number results are summarized in Figure 4, where it is possible to observe that the highest average normalized score was obtained for PDB code 1ENY, which refers to enoyl‐acyl carrier protein reductase, involved in mycolic acid synthesis. Among the 10 best ranked targets (overall average > 0.80), five other PDB entries also refer to enoyl‐acyl carrier protein reductase (4BGI, 2IE0, 4BGE, 2H9I, and 4BII), while 3R5L corresponds to deazaflavin‐dependent nitroreductase, which also acts in mycolic acid synthesis. Only 1EYE, 1XFC, and 5UHG are not involved in this pathway.

Regarding the compounds under study, it is important to highlight that, in the four scoring functions, compound A17 presented the highest score of all compounds, either with PDB code 1ENY in ASP, ChemPLP, and ChemScore, or with the 2IE0 entry in GoldScore. Both codes correspond to Enoyl‐acyl carrier protein reductase, indicating that a high‐affinity complex can be formed between this enzyme and ligand A17. This result agrees with the in vitro tests displayed in Table 2, which have demonstrated that this is the most potent compound against the MTB strains tested.

**FIGURE 5:**
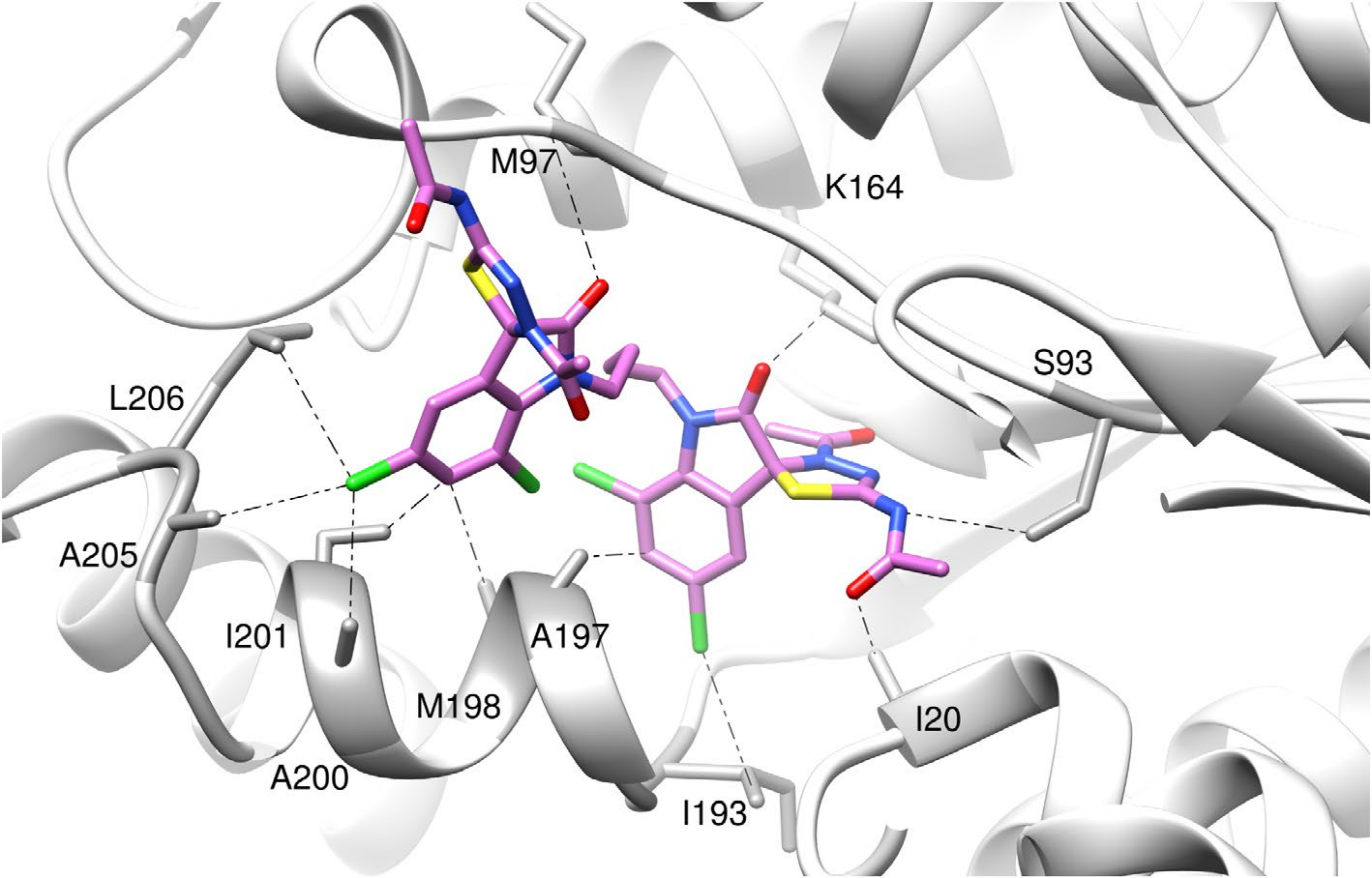
Main interactions between ligand A17_1S,2S and target 1ENY, which yielded the highest docking score using the ASP scoring function. The protein is depicted in gray, and ligand A17_1S,2S in pink. Heteroatoms are represented in distinct colors: red for oxygen, green for chlorine, blue for nitrogen, and yellow for sulfur.

Figure 5 shows the main ligand‐receptor interactions, where it is possible to observe that hydrogen bonds are established with residues I20, S93, M97, and K164, while a halogen bond is formed by one of the chlorine atoms and I193. These are key interactions observed in the crystallographic structure with the ligands in PDB codes 1ENY and 6SQ7. A hydrophobic pocket composed of residues A197, M198, A200, I201, A205, and L206 accommodates the other spirooxindole monomer, which is the same found by Kuldeep et al. (2021) when docking a hit molecule obtained from a virtual screening study against MTB Enoyl‐acyl carrier protein reductase.

The structure–activity relationship (SAR) analysis of the 17 derivatives revealed meaningful correlations between structural substitutions and the observed antimycobacterial activity. Compounds A16 and A17, which exhibited the lowest minimum inhibitory concentrations (MICs) against both drug‐sensitive and multidrug‐resistant *Mycobacterium tuberculosis* strains (10–20 μM), share common structural features that appear to be associated with enhanced biological activity.

Both A16 and A17 contain chlorine atoms as substituents on their aromatic rings, which may promote hydrophobic interactions within the active site of the target enzyme. In contrast, compounds A1 through A14, which showed no significant activity (MIC > 200 μM), lack these halogen substitutions. Additionally, the analysis of stereoisomers indicates that stereochemistry influences the binding affinity to the active site. For example, the configuration of compound A17_1S_2S yielded the highest docking scores, likely due to a more favorable spatial orientation of functional groups capable of forming hydrogen bonds with residues such as S93 and K164, as well as deep hydrophobic interactions with residues A197 and L206. A halogen bond was also observed between the chlorine atom and residue I193.

On the other hand, stereoisomers with meso (1S_2R) or 1R_2R configurations exhibited slightly lower docking scores, possibly indicating less favorable orientations for active site binding. Furthermore, comparing A15, A16, and A17 reveals that, although they share the same spiro‐isatin core, modifications to the aromatic substituents and side chains directly affect molecular affinity. The presence of chlorine atoms in A16 and A17 seems to be linked to increased lipophilicity and halogen bonding capacity, which in turn enhances target binding.

Enoyl‐acyl carrier protein reductases (ENRs) are a group of enzymes that catalyze the final step in the fatty acid biosynthesis pathway, namely the reduction of the substrate enoyl thioester to an acyl moiety (Ghattas et al. 2016). The main function of this enzyme is in the final step of fatty acid biosynthesis, which involves the reduction of 2‐trans‐enoyl‐acyl carrier protein by utilizing NADH in the elongation step of the FASII pathway (Vilchéze et al. 2000). ENR has been validated as a potential antimalarial and antibacterial drug target (Chhibber et al. 2006). The role of enoyl‐acyl carrier protein reductase as one of the best‐verified targets for the development of antituberculosis drugs has been recently reviewed by Prasad et al. (2021), with several scaffolds already identified with the aid of high‐throughput screening, encoded library technology, and also fragment‐based screening, spanning different classes of molecules. Known inhibitors so far are classified as indirect inhibitors (Isoniazid, Ethionamide, Prothionamide) and direct inhibitors (Triclosan/Diphenyl ethers, Pyrrolidine Carboxamides, Pyrroles, Acetamides, Thiadiazoles, Triazoles). To date, our work is the first evidence that spiro 1,3,4‐thiadiazolines may act as inhibitors of this enzyme. Due to their size and shape, the docking results suggest that these ligands may compete with NADH for the binding site, potentially acting as competitive inhibitors. Therefore, future enzymatic assays should take into account the influence of NADH concentration to determine whether the ligand exhibits competitive inhibition.

### 3.5 Evaluation of the Docking Protocol

Table 3 presents the RMSD values calculated for the four different GOLD scoring functions. Since the lowest RMSD was obtained with the ASP scoring function, using the 4OHU PDB code, the further analyses were performed using this approach.

**TABLE 3:** Root mean square deviations obtained in the redocking tests for the four GOLD scoring functions.

GOLD scoring function	RMSD (Å)
ASP	0.24
ChemScore	2.74
ChemPLP	1.64
Goldscore	1.18

The ROC Curve (Receiver Operating Characteristic) is a two‐dimensional graphical tool used to evaluate the accuracy of docking protocols, as it provides information about data sensitivity and specificity (Hsu et al. 2014). It is plotted with true positives on the *y*‐axis and false positives on the *x*‐axis. The closer the value of the area under the curve (AUC) approaches the number 1, the better the quality of the protocol and its ability to discriminate between the analyzed groups (generally divided between active and inactive).

**FIGURE 6:**
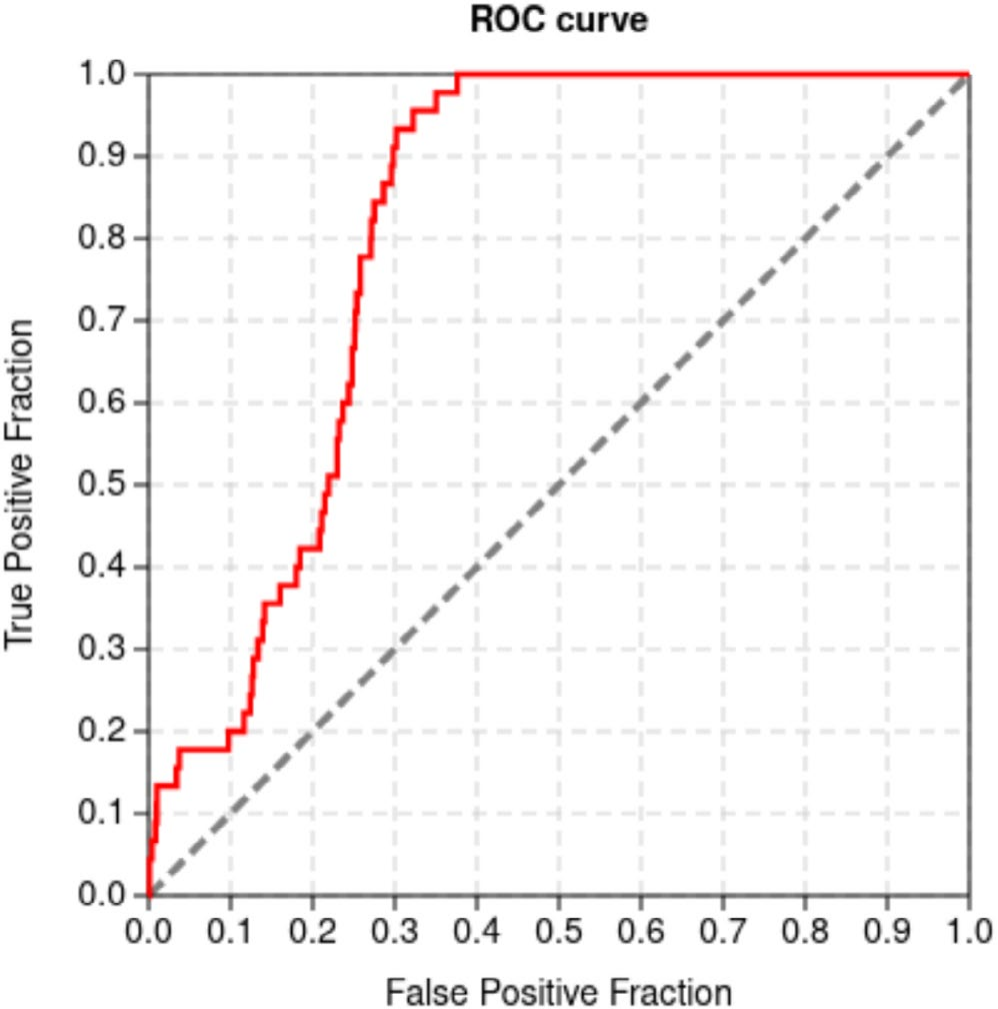
ROC curve for docking the molecules from the data set into the structure of the enzyme enoyl‐[acyl carrier protein] reductase (PDB code 4OHU) in the GOLD program with the ASP function.

Additional evidence of the accuracy of the docking protocol provided by the GOLD program was thus obtained by the ROC curve. For this, the dataset of ligands was classified into three categories: the active ones, which presented the better experimental results, that is, low *K*_i_ values; the inactive ones, with higher *K*_i_ results; and the decoys. By docking the 2099 molecules in the GOLD program with the ASP score function for these three classes of compounds, active, inactive, and decoys, the results of each score obtained for each predicted pose were used in the statistical analysis of the ROC curve. To analyze the points on the curve in Figure 6, the effect size indicator for ROC curves used was the AUC (area under curve), the result of the integration of all points on the curve.

The Area Under the Curve (AUC) serves as an effective metric for evaluating the performance of an in silico testing protocol. An AUC value near 0.5 indicates a test with performance comparable to random chance, while a value of 1 represents an ideal outcome (Triballeau et al. 2005). For the evaluated data, the AUC was 0.812, suggesting a probability of correct classification in 81% of cases, indicating a high capacity of the docking protocol used to distinguish active and inactive compounds.

### 3.6 Characterization of the Pharmacokinetic Profile

The pharmacokinetic properties of the ligands under study, A15, A16, and A17, are displayed in Table 4. For this case, there was no need to differentiate the chiral centers, as the online servers SwissADME and pkSCM do not consider the 3D conformations of the molecules.

**TABLE 4:** Pharmacokinetic properties calculated for the ligands under study.

Property	Molecule		
	A15	A16	A17
Water solubility	Moderate	Low	Low
Log P	2.41	3.39	3.74
Molecular weight (g mol^−1^)	717.60	789.49	800.52
Number of hydrogen acceptors	8	8	8
Number of hydrogen donors	2	2	2
BBB	No	No	No
AMES toxicity	Yes	No	No
GI absorption (%)	92.50	94.79	92.53
Protein inhibition	CYP3A4	CPY2C19	No
P‐gp	Yes	Yes	Yes

Regarding the parameters associated with Lipinski's rule (molecular weight, Log P, number of hydrogen bond acceptors and number of hydrogen bond donors), it can be observed that all compounds present acceptable oral bioavailability (Baca et al. 2000). The exception is the violation for molecular weight, since values higher than 500 g/mol were obtained, although this rule has been questioned, especially in the context of antibiotic drug discovery (Blaskovich et al. 2015). However, the number of hydrogen bond acceptors did not exceed the limit of 10, while donors did not exceed the limit of 5. Although water solubility is predicted as low for A16 and A17, their log P values are lower than 5, showing acceptable lipophilicity and permeability of the molecules in the membrane. None of the molecules exhibit BBB permeability, suggesting limited central nervous system (CNS) penetration. This could be beneficial for reducing CNS‐related side effects if the target is non‐CNS related.

The AMES toxicity, a parameter that is related to the capacity of a molecule to generate genetic mutations in living beings (Galbán and Duckett 2010), pkSCM indicates that only A15 is predicted to be mutagenic, while A16 and A17 do not exhibit this risk, indicating good potential for oral bioavailability despite their high molecular weights. Regarding metabolic interactions, A15 is predicted to inhibit CYP3A4, while A16 may inhibit CYP2C19, and A17 does not inhibit these enzymes. This suggests that A17 may have a lower potential for drug–drug interactions via cytochrome P450 inhibition. All three molecules are predicted to be substrates for P‐glycoprotein (P‐gp), which plays a role in drug efflux and can impact absorption, distribution, and resistance mechanisms, which may suggest further experimental tests to confirm these results, and also structural modifications, since high P‐gp interaction may reduce intracellular drug accumulation, potentially affecting efficacy.

## 4 Conclusions

In this study, a series of 17 isatin derivatives were evaluated for their antituberculosis activity against *Mycobacterium tuberculosis* (MTB) strains, including multidrug‐resistant clinical isolates. Among them, compounds A16 and A17 exhibited the highest potency, with molecular docking studies suggesting enzyme enoyl‐[acyl‐carrier‐protein] reductase as a potential biological target. Additionally, cytotoxicity assays confirmed that compounds A16 and A17 are non‐toxic to mammalian cells, reinforcing their therapeutic potential. Computational ADMET analysis further indicated that these compounds possess favorable pharmacokinetic and pharmacodynamic properties for anti‐TB drug development. Importantly, A15, A16, and A17 were tested against the multidrug‐resistant MTB clinical isolates PT‐12 and PT‐20, which carry mutations associated with rifampicin (*rpoB* S531L) and isoniazid (*katG* S315T) resistance. A16 and A17 exhibited MIC values ranging from 10 to 20 μM, significantly lower than those of isoniazid (146–292 μM) and rifampicin (> 80 μM), suggesting that these compounds may overcome key resistance mechanisms. Overall, our findings highlight A16 and A17 as promising lead compounds for further optimization and experimental validation, particularly in the fight against multidrug‐resistant tuberculosis.

## Conflicts of Interest

The authors declare no conflicts of interest.

## Supporting information


Data S1.

Data S2.

## Data Availability

The data that supports the findings of this study are available in the supporting information of this article.

## References

[cbdd70152-bib-0001] Baca, A. M. , R. Sirawaraporn , S. Turley , W. Sirawaraporn , and W. G. Hol . 2000. “Crystal Structure of *Mycobacterium tuberculosis* 6‐Hydroxymethyl‐7, 8‐Dihydropteroate Synthase in Complex With Pterin Monophosphate: New Insight Into the Enzymatic Mechanism and Sulfa‐Drug Action.” Journal of Molecular Biology 302, no. 5: 1193–1212. 10.1006/jmbi.2000.4094. 11007651

[cbdd70152-bib-0002] Berman, H. M. , J. Westbrook , Z. Feng , et al. 2000. “The Protein Data Bank.” Nucleic Acids Research 28, no. 1: 235–242. 10.1093/nar/28.1.235. 10592235 PMC102472

[cbdd70152-bib-0003] Blaskovich, M. A. , J. Zuegg , A. G. Elliott , and M. A. Cooper . 2015. “Helping Chemists Discover New Antibiotics.” ACS Infectious Diseases 1, no. 7: 285–287. 10.1021/acsinfecdis.5b00044. 27622818

[cbdd70152-bib-0004] Blumenthal, G. M. , P. Cortazar , J. J. Zhang , et al. 2012. “FDA Approval Summary: Sunitinib for the Treatment of Progressive Well‐Differentiated Locally Advanced or Metastatic Pancreatic Neuroendocrine Tumors.” Oncologist 17, no. 8: 1108–1113. 10.1634/theoncologist.2012-0044. 22836448 PMC3425529

[cbdd70152-bib-0005] Castro, A. , I. M. G. Andrade , M. C. Coelho , et al. 2023. “Multicomponent Synthesis of Spiro 1, 3, 4‐Thiadiazolines With Anticancer Activity by Using Deep Eutectic Solvent Under Microwave Irradiation.” Journal of Heterocyclic Chemistry 60, no. 3: 392–405. 10.1002/jhet.4591.

[cbdd70152-bib-0006] Cheke, R. S. , V. M. Patil , S. D. Firke , et al. 2022. “Therapeutic Outcomes of Isatin and Its Derivatives Against Multiple Diseases: Recent Developments in Drug Discovery.” Pharmaceuticals 15, no. 3: 272. 10.3390/ph15030272. 35337070 PMC8950263

[cbdd70152-bib-0007] ChemAxon . 2023. “ChemAxon Marvin 23.10.” https://www.chemaxon.com.

[cbdd70152-bib-0008] Chen, V. B. , W. B. Arendall , J. J. Headd , et al. 2010. “MolProbity: All‐Atom Structure Validation for Macromolecular Crystallography.” Acta Crystallographica, Section D: Biological Crystallography 66, no. 1: 12–21. 10.1107/S0907444909042073. 20057044 PMC2803126

[cbdd70152-bib-0009] Chhibber, M. , G. Kumar , P. Parasuraman , T. N. C. Ramya , N. Surolia , and A. Surolia . 2006. “Novel Diphenyl Ethers: Design, Docking Studies, Synthesis and Inhibition of Enoyl ACP Reductase of Plasmodium Falciparum and *Escherichia coli* .” Bioorganic & Medicinal Chemistry 14, no. 23: 8086–8098. 10.1016/j.bmc.2006.07.034. 16893651

[cbdd70152-bib-0010] da Costa, D. P. , A. C. de Castro , G. A. da Silva , et al. 2021. “Microwave‐Assisted Synthesis and Antimicrobial Activity of Novel Spiro 1, 3, 4‐Thiadiazolines From Isatin Derivatives.” Journal of Heterocyclic Chemistry 58, no. 3: 766–776. 10.1002/jhet.4213.

[cbdd70152-bib-0011] Daina, A. , O. Michielin , and V. Zoete . 2017. “SwissADME: A Free Web Tool to Evaluate Pharmacokinetics, Drug‐Likeness and Medicinal Chemistry Friendliness of Small Molecules.” Scientific Reports 7: 42717. 10.1038/srep42717. 28256516 PMC5335600

[cbdd70152-bib-0013] Dhanoa, R. , J. S. Delaney , and J. Karanicolas . 2011. “H++: A Web‐Based Server for Predicting Protein Protonation States.” Bioinformatics 27, no. 17: 2361–2362. 10.1093/bioinformatics/btr374.21752801

[cbdd70152-bib-0014] Dheda, K. , F. Mirzayev , D. M. Cirillo , et al. 2024. “Multidrug‐Resistant Tuberculosis.” Nature Reviews Disease Primers 10, no. 1: 22. 10.1038/s41572-024-00504-2. PMC13335523 38523140

[cbdd70152-bib-0015] Galati, S. , C. R. Andrade , and D. F. Costa . 2021. “Recent Advances in In Silico Target Fishing.” Molecules 26, no. 17: 5124. 10.3390/molecules26175124. 34500568 PMC8433825

[cbdd70152-bib-0016] Galbán, S. , and C. S. Duckett . 2010. “XIAP as a Ubiquitin Ligase in Cellular Signaling.” Cell Death and Differentiation 17, no. 1: 54–60. 10.1038/cdd.2009.129. 19590513 PMC2957808

[cbdd70152-bib-0017] Gaulton, A. , A. Hersey , L. Bastarache , D. Ochoa , and A. Williams . 2012. “ChEMBL: A Large‐Scale Bioactivity Database for Drug Discovery.” Nucleic Acids Research 40, no. 1: 1100–1107. 10.1093/nar/gkr777. PMC3245175 21948594

[cbdd70152-bib-0018] Ghattas, M. A. , R. A. Mansour , N. Atatreh , and R. A. Bryce . 2016. “Analysis of Enoyl‐Acyl Carrier Protein Reductase Structure and Interactions Yields an Efficient Virtual Screening Approach and Suggests a Potential Allosteric Site.” Chemical Biology & Drug Design 87, no. 1: 131–142. 10.1111/cbdd.12635. 26259619

[cbdd70152-bib-0019] Hsu, M. J. , Y. C. I. Chang , and H. M. Hsueh . 2014. “Biomarker Selection for Medical Diagnosis Using the Partial Area Under the ROC Curve.” BMC Research Notes 7, no. 1: 1–15. 10.1186/1756-0500-7-15. 24410929 PMC3923449

[cbdd70152-bib-0020] Jiang, D. , G. Q. Wang , X. Liu , Z. Zhang , L. S. Feng , and M. L. Liu . 2018. “Isatin Derivatives With Potential Antitubercular Activities.” Journal of Heterocyclic Chemistry 55, no. 6: 1263–1279. 10.1002/jhet.3189.

[cbdd70152-bib-0021] Jones, G. , P. Willett , R. C. Glen , A. R. Leach , and R. Taylor . 1997. “Development and Validation of a Genetic Algorithm for Flexible Docking.” Journal of Molecular Biology 267, no. 3: 727–748. 10.1006/jmbi.1996.0897. 9126849

[cbdd70152-bib-0022] Kuhn, M. , and T. Sander . 2013. “The Drug Design Statistics Platform.” European Journal of Medicinal Chemistry 63: 74–82. 10.1016/j.ejmech.2013.02.006.

[cbdd70152-bib-0023] Kuldeep, J. , S. K. Sharma , T. Sharma , B. N. Singh , and M. I. Siddiqi . 2021. “Targeting *Mycobacterium tuberculosis* Enoyl‐Acyl Carrier Protein Reductase Using Computational Tools for Identification of Potential Inhibitor and Their Biological Activity.” Molecular Informatics 40, no. 5: 2000211. 10.1002/minf.202000211. 33283460

[cbdd70152-bib-0024] London, C. A. , P. B. Malpas , S. L. Wood‐Follis , et al. 2009. “Multi‐Center, Placebo‐Controlled, Double‐Blind, Randomized Study of Oral Toceranib Phosphate (SU11654), a Receptor Tyrosine Kinase Inhibitor, for the Treatment of Dogs With Recurrent (Either Local or Distant) Mast Cell Tumor Following Surgical Excision.” Clinical Cancer Research 15, no. 11: 3856–3865. 10.1158/1078-0432.CCR-08-1860. 19470739

[cbdd70152-bib-0025] Mysinger, M. M. , and B. K. Shoichet . 2010. “Rapid Context‐Dependent Ligand Desolvation in Molecular Docking.” Journal of Chemical Information and Modeling 50, no. 4: 1561–1573. 10.1021/ci100050v. 20735049

[cbdd70152-bib-0026] Nalini, R. , S. M. Basavarajaiah , G. Y. Nagesh , J. Mohammad , and K. Ramakrishna Reddy . 2023. “Synthesis, Characterization, DFT Analysis, Biological Evaluation, and Molecular Docking of Schiff Base Derived From Isatin–Isoniazid and Its Metal (II) Complexes.” Polycyclic Aromatic Compounds 43, no. 8: 7597–7614. 10.1080/10406638.2022.2138927.

[cbdd70152-bib-0027] Nath, R. , S. Pathania , G. Grover , and M. J. Akhtar . 2020. “Isatin Containing Heterocycles for Different Biological Activities: Analysis of Structure Activity Relationship.” Journal of Molecular Structure 1222: 128900. 10.1016/j.molstruc.2020.128900.

[cbdd70152-bib-0028] O'Boyle, N. M. , M. Banck , C. A. James , C. Morley , T. Vandermeersch , and G. R. Hutchison . 2011. “Open Babel: An Open Chemical Toolbox.” Journal of Cheminformatics 3: 33. 10.1186/1758-2946-3-33. 21982300 PMC3198950

[cbdd70152-bib-0029] Perdigão, J. , H. Silva , D. Machado , et al. 2014. “Unraveling *Mycobacterium tuberculosis* Genomic Diversity and Evolution in Lisbon, Portugal, a Highly Drug‐Resistant Setting.” BMC Genomics 15: 991. 10.1186/1471-2164-15-991. 25407810 PMC4289236

[cbdd70152-bib-0030] Pettersen, E. F. , T. D. Goddard , C. C. Huang , et al. 2004. “UCSF Chimera—A Visualization System for Exploratory Research and Analysis.” Journal of Computational Chemistry 25, no. 13: 1605–1612. 10.1002/jcc.20084. 15264254

[cbdd70152-bib-0031] Pires, D. E. , T. L. Blundell , and D. B. Ascher . 2015. “pkCSM: Predicting Small‐Molecule Pharmacokinetic Properties Using Graph‐Based Signatures.” Journal of Medicinal Chemistry 58, no. 9: 4066–4072. 10.1021/acs.jmedchem.5b00213. 25860834 PMC4434528

[cbdd70152-bib-0032] Popat, S. , A. Mellemgaard , K. Fahrbach , et al. 2015. “Nintedanib Plus Docetaxel as Second‐Line Therapy in Patients With Non‐Small‐Cell Lung Cancer: A Network Meta‐Analysis.” Future Oncology 11, no. 3: 409–420. 10.2217/fon.14.290. 25478720

[cbdd70152-bib-0033] Prasad, M. S. , R. P. Bhole , P. B. Khedekar , and R. V. Chikhale . 2021. “Mycobacterium Enoyl Acyl Carrier Protein Reductase (InhA): A Key Target for Antitubercular Drug Discovery.” Bioorganic Chemistry 115: 105242. 10.1016/j.bioorg.2021.105242. 34392175

[cbdd70152-bib-0034] Richeldi, L. , R. M. Du Bois , G. Raghu , et al. 2014. “Efficacy and Safety of Nintedanib in Idiopathic Pulmonary Fibrosis.” New England Journal of Medicine 370, no. 22: 2071–2082. 10.1056/nejmoa1402584. 24836310

[cbdd70152-bib-0035] Santos, D. M. , A. L. Ferreira , J. C. Souza , et al. 2023. “Antiproliferative Activity, Preliminary QSAR Analysis and In Silico ADME Studies of Morita‐Baylis‐Hillman Adducts From Isatin Derivatives in Four Cancer Cell Lines.” Revista Virtual de Química 15, no. 1: 3–11. https://www.scielo.br/j/jbchs/a/DJcvtMb8RHxnF8jcMLqQJtk/?format=pdf&lang=en.

[cbdd70152-bib-0037] Singh, G. , A. Kumar , P. Mann , and J. Kaur . 2017. “Cell Wall Factors of *Mycobacterium tuberculosis* as Major Virulence Determinants: Current Perspectives in Drugs Discovery and Design.” Betham Science 18, no. 1: 1904–1918. 10.2174/1389450118666170711150034. 28699515

[cbdd70152-bib-0038] Singh, G. S. , and Z. Y. Desta . 2012. “Isatins as Privileged Molecules in Design and Synthesis of Spiro‐Fused Cyclic Frameworks.” Chemical Reviews 112, no. 11: 6104–6155. 10.1021/cr300135y. 22950860

[cbdd70152-bib-0039] Sridhar, B. T. , G. Y. Nagesh , K. Prashantha , et al. 2024. “Discovery of Novel Isatin Encompassing Oxadiazoles as Potential Inhibitors Against New Delhi Metallo‐β‐Lactamase‐1: Synthesis, Spectral Analysis, Antimicrobial, and Molecular Modeling Studies.” Russian Journal of Bioorganic Chemistry 50, no. 4: 1376–1389. 10.1134/S106816202404023X.

[cbdd70152-bib-0041] The CRyPTIC Consortium . 2022. “A Data Compendium Associating the Genomes of 12,289 *Mycobacterium tuberculosis* Isolates With Quantitative Resistance Phenotypes to 13 Antibiotics.” PLoS Biology 20: e3001721. 10.1371/journal.pbio.3001721. 35944069 PMC9363010

[cbdd70152-bib-0042] Triballeau, N. , F. Acher , I. Brabet , J. P. Pin , and H. O. Bertrand . 2005. “Virtual Screening Workflow Development Guided by the “Receiver Operating Characteristic” Curve Approach. Application to High‐Throughput Docking on Metabotropic Glutamate Receptor Subtype 4.” Journal of Medicinal Chemistry 48, no. 7: 2534–2547. 10.1021/jm049092j. 15801843

[cbdd70152-bib-0043] Vilchéze, C. , H. R. Morbidoni , T. R. Weisbrod , et al. 2000. “Inactivation of the inhA‐Encoded Fatty Acid Synthase II (FASII) Enoyl‐Acyl Carrier Protein Reductase Induces Accumulation of the FASI End Products and Cell Lysis of *Mycobacterium smegmatis* .” Journal of Bacteriology 182, no. 14: 4059–4067. 10.1128/JB.182.14.4059-4067.2000. 10869086 PMC94593

[cbdd70152-bib-0044] Wang, R. , and S. Wang . 2001. “How Does Consensus Scoring Work for Virtual Library Screening? An Idealized Computer Experiment.” Journal of Chemical Information and Computer Sciences 41, no. 5: 1422–1426. 10.1021/ci0100567. 11604043

[cbdd70152-bib-0045] Waterhouse, A. , M. Bertoni , S. Bienert , et al. 2018. “SWISS‐MODEL: Homology Modelling of Protein Structures and Complexes.” Nucleic Acids Research 46, no. 1: 296–303. 10.1093/nar/gky427. PMC6030848 29788355

[cbdd70152-bib-0046] World Health Organization . 2024. “Global Tuberculosis Report 2024.” https://www.who.int/publications/i/item/9789240101531.

[cbdd70152-bib-0047] World Health Organization . 2025. WHO Consolidated Operational Handbook on Tuberculosis. Module 4: Treatment and Care. World Health Organization. https://www.who.int/publications/i/item/9789240108141.

[cbdd70152-bib-0048] Yu, W. , and A. D. Mackerell . 2017. “Métodos de Design de Medicamentos Auxiliados por Computador.” In Antibióticos. Métodos em Biologia Molecular, edited by P. Sass , vol. 1520, 67–84. Humana Press. 10.1007/978-1-4939-6634-9_5.

[cbdd70152-bib-0049] Ziegler, S. , S. Caputo , G. Tóth , et al. 2010. “Target Identification for Small Bioactive Molecules: Finding the Needle in the Haystack.” Angewandte Chemie International Edition 52, no. 10: 2744–2792. 10.1002/anie.201208749. 23418026

