# Supplementary material for: In Vitro and In Silico Evaluation of Isatin‐Derived Spirooxindoles as Antituberculosis Drug Candidates
Source: Chem Biol Drug Des. 2025 Jul 1;106(1):e70152. doi: 10.1111/cbdd.70152 (PMC12212626; doi:10.1111/cbdd.70152)
Supplement: Supplementary file 1 — Data S1. [file CBDD-106-e70152-s002.docx]

**Supporting Information to**

***In vitro* and *in silico* evaluation of isatin-derived spirooxindoles as antituberculosis drug candidates**

Fernanda Rodrigues de Lima^a^, Jéssika de Oliveira Viana^a,b^, Aleff Cruz de Castro^a^, Rodrigo Cristiano^a,b^, Marcia Alberton Perelló^c^, Alexia de Matos Czeczot^c,d^, Cristiano Valim Bizarro^c,d^, Pablo Machado^c,d,e^, Luiz Augusto Basso^c,d,e^, Claudio Gabriel Lima-Junior^a,b^, Valnês da Silva Rodrigues-Junior^f,*^*,* Karen Cacilda Weber*^a,b^*^*^

**1 Screening for anti-mycobacterial potential**

Anti-mycobacterial activity was determined by using the resazurin reduction microplate assay (REMA) as a growth indicator (Taneja & Tyagi 2007). *M. tuberculosis* H37Ra suspensions were grown in Middlebrook 7H9, with 10% OADC (oleic acid, albumin, dextrose, catalase; Bec-ton Dickinson), 0.2% glycerol, and 0.05% Tween-80. Test compounds were first solubilized in dimethyl sulfoxide (DMSO) at a concentration of 16 mM, and then diluted in Middlebrook 7H9 + 10% OADC broth to reach a concentration of 200 µM. Serial two-fold dilutions were performed in 96-well U-bottom polystyrene microplates at concentration ranges of 160-1.25 µM for all compounds. DMSO concentration was maintained at 2.5% in all experimental groups. Mycobacterial suspensions were diluted in 7H9 medium at an optical density (OD595nm, WPA UVA 1101 Biotech Photometer – Gemini BV) of 0.006, and 100 µL were added to each well. Following incubation at 37 °C for 7 days, 30 µL of a sterile resazurin solution (0.02%) were added to the plates and the color (blue or pink) of each well was visually read after 48 hours (Sidrônio et al. 2021). Minimum inhibitory concentrations (MICs) were considered as the lowest compound concentration that prevented a color change from blue (resazurin) to pink (resorufin). Rifampicin and moxifloxacin were used as positive control drugs.

**2 Determination of MIC against virulent and resistant *M. tuberculosis***

Drugs that exhibited bacterial growth inhibition at MICs lower than 50 µM were chosen for additional testing against the drug-sensitive H37Rv strain and multi-drug resistant clinical isolates of *M. tuberculosis*, PT-12 and PT-20. The clinical isolates used in this work have been previously characterized by Perdigão et al. (2014). MDR PT-12 and PT-20 carry the mutation (S531L) in the *rpo*B gene, responsible for causing resistance to rifampicin and also carry the mutation (S315T) in the *kat*G (Rv1908c) gene, which is the most frequent mutation found in isoniazid-resistant strains. MICs were determined by using the REMA method. Mycobacterial suspensions cultivation, assay procedures, and result analysis were performed as previously described before, in section screening for anti-mycobacterial potential (Taneja & Tyagi 2007; Sidrônio et al. 2021).

**3 Cytotoxicity investigation**

The lead compounds A16 and A17 were selected for toxicity investigations. Cellular viability determination after incubation with the test compound was performed as described by De Sousa et al. 2025. African green monkey kidney (Vero) cells, obtained from Cell Bank of Rio de Janeiro, were cultured in DMEM media (Dulbecco's Modified Eagle Medium) supplemented with 10% inactivated fetal bovine serum (FBS) and 1% antibiotics (penicillin-streptomycin). The cells were maintained in culture flasks at 37 °C in a humidified atmosphere with 5% CO2. Cells were seeded at 5 x 10^3^ and incubated overnight to adhere. The following day, the cellular samples underwent treatment with solutions of A16 and A17, resulting in concentrations ranging from 12.5 to 100 µM (DMSO 1 %, v/v). After 72 h at 37 °C under 5 % of CO_2_, all wells received MTT solution (0.5 mg/mL) for 3 h. The formazan crystals were dried overnight at room temperature and dissolved in DMSO. The optical density was recorded at 570 nm (Absorbance microplate reader EL800, BioTek, USA). The percentages of cell viability for treated groups were reported considering the control wells (DMSO 1 %-treated) as 100 % of cell viability. Data were expressed as mean of cell viability ± standard error of mean of three-four independent experiments performed in triplicates. The statistical analysis was performed by one-way analysis of variance, followed by Bonferroni’s post-test, using GraphPad Prism 8.0 (San Diego, CA, USA). Differences were considered significant at the 95 % level of confidence.

**References**

De Sousa, N. F., de Freitas, M. E. G., Sidrônio, M. G. S., Souza, H. D., Czeczot, A., Perelló, M., Fiss, G. F., Scotti, L., de Araújo, D. A. M., Barbosa Filho, J. M., et al. (2025). Preclinical evaluation of selene-ethylenelacticamides in tuberculosis: effects against active, dormant, and resistant Mycobacterium tuberculosis and in vitro toxicity investigation. Microorganisms, 13, 396. <https://doi.org/10.3390/microorganisms13020396>

Perdigão, J., Silva, H., Machado, D., Macedo, R., Maltez, F., Silva, C., Jordao, L., Couto, I., Mallard, K., Coll, F., Hill-Cawthorne, G., McNerney, R., Pain, A., Clark, T. G., Viveros, M., & Portugal, I. (2014). Unraveling Mycobacterium tuberculosis genomic diversity and evolution in Lisbon, Portugal, a highly drug-resistant setting. BMC Genomics, 15, 991. <https://doi.org/10.1186/1471-2164-15-991>

Sidrônio, M. G. S., Castelo Branco, A. P. O. T., Abbadi, B. L., Macchi, F., Silveira, M. D., Lock, G. A., Rodrigues-Junior, V. S. (2021). Effects of tafenoquine against active, dormant, and resistant Mycobacterium tuberculosis. Tuberculosis, 128, 102089. <https://doi.org/10.1016/j.tube.2021.102089>

Taneja, N. K., & Tyagi, J. S. (2007). Resazurin reduction assays for screening of anti-tubercular compounds against dormant and actively growing Mycobacterium tuberculosis, Mycobacterium bovis BCG, and Mycobacterium smegmatis. Journal of Antimicrobial Chemotherapy, 60(2), 288–293. <https://doi.org/10.1093/jac/dkm207>
